# Haptic Feedback Reduces Telesurgery Operators’ Reaction Times Compared to Conventional Stimulation: Results of a First-in-Human Study

**DOI:** 10.3390/s26092597

**Published:** 2026-04-23

**Authors:** Vaidas Labunskas, Vilius Dambrauskas, Augustė Melaikaitė, Vilhelmas Konstantinas Landsbergis, Radvilė Kadytė, Augustinas Baušys, Tomas Baltrūnas

**Affiliations:** 1 Health Telematics Science Institute, Kaunas University of Technology, 44249 Kaunas, Lithuania; vilius.dambrauskas@ktu.edu; 2 Inovatyvi Medicina, UAB, 44352 Kaunas, Lithuania; 3 Faculty of Medicine, Vilnius University, 01513 Vilnius, Lithuania; auguste.melaikaite@mf.stud.vu.lt (A.M.); vilhelmas.landsbergis@mf.stud.vu.lt (V.K.L.); radvile.kadyte@mf.stud.vu.lt (R.K.); 4 Laboratory of Experimental Surgery and Oncology, Faculty of Medicine, Vilnius University, 01513 Vilnius, Lithuania; augustinas.bausys@mf.vu.lt

**Keywords:** haptic feedback, reaction time, telesurgery, latency, delay, robotic surgery

## Abstract

This prospective, cross-sectional study evaluated reaction time (RT) variations across different sensory stimuli to investigate the efficacy of haptic feedback (HF) in reducing response latency for telesurgical applications. Three healthy-volunteer age cohorts (18–25, 35–45, and 55–65 years) were tested using visual, auditory, superficial, and deep sensations, alongside a multimodal stimulus combining visual and superficial inputs to simulate HF. The findings revealed that combined visual and superficial stimulation yielded a mean RT of 227 ± 27 ms, outperforming visual-only stimulation by 40 ms (95% CI: 32–48 ms) and superficial-only stimulation by 26 ms (95% CI: 20–33 ms) (*p* = 0.001). While this performance boost was consistent across all age groups, the 55–65 age cohort demonstrated the most pronounced reduction in RT when the combined stimuli were used. These results suggest that integrating tactile sensations with visual cues significantly mitigates latency compared to unimodal inputs, underscoring the potential of haptic feedback to enhance operator performance and safety in latency-sensitive environments like remote surgery.

## 1. Introduction

Telesurgery represents a transformative paradigm in modern medicine, leveraging high-speed wireless communication networks and robotic systems to allow surgeons to operate on patients across vast geographical distances [1]. The feasibility of such systems is enhanced by mechanical master–slave architectures, which enable precise one-to-one mapping between the surgeon’s movements on an ergonomic master device and the slave robotic arms at the surgical site [2]. Since the landmark 2001 “Operation Lindbergh”—where surgeons in New York performed a remote robotic cholecystectomy on a patient in Strasbourg—the feasibility of long-distance surgery has been well-established [3]. By eliminating the need for physical proximity, telesurgery offers a solution to several systemic challenges in healthcare: it facilitates expert surgical care in underserved regions, minimizes involuntary oscillations through robotic filtering, and optimizes the distribution of specialized medical resources [4,5]. The clinical necessity of such remote interventions is perhaps most acute in the treatment of ischemic stroke. In the United States, approximately 335,000 individuals annually suffer from ischemic strokes caused by large-vessel occlusions (LVOs). While mechanical thrombectomy is the established standard of care, it remains a drastically underutilized intervention. As of 2022, only 311 centers in the United States were capable of performing thrombectomies, resulting in a stark geographic disparity where 50% of the population resides more than one hour away from a capable facility. Consequently, only about 10% of LVO patients currently undergo this necessary procedure. This delay is catastrophic: every 10-min lag in revascularization reduces a patient’s disability-free life by approximately 40 days and incurs an additional $10,000 in healthcare costs. Although the therapeutic window has expanded to 24 h in some cases, the “time is brain” principle dictates that remote, robotic intervention could bridge the gap between rural patients and reception of life-saving specialized care [6]. Despite this potential, widespread adoption of telesurgery is hindered by several technical and organizational barriers, the most notable of which is network latency. Latency—the delay in data transmission—can impair surgical precision, prolong operative times, and increase the risk of procedural errors [7]. Research indicates that telesurgical performance declines exponentially as latency increases [8], while a threshold of 100 to 200 ms is generally considered the upper limit for most procedures [8,9,10]. Complex laparoscopic tasks may show degradation at just 100 ms, whereas endovascular procedures, such as thrombectomy, may remain viable with delays up to 400 ms [11,12,13,14]. At higher latencies, increased instrument displacement and surgeon fatigue become significant risks to patient safety [8,9,10,11,12,14]. A promising avenue for mitigating the effects of latency involves the integration of multi-sensory feedback. Traditional robotic platforms rely almost exclusively on visual data, depriving the surgeon of the haptic feedback (HF) found in conventional surgery. Currently, only select systems, such as the Da Vinci 5 and Sentante, offer haptic capabilities [15,16]. Preliminary studies suggest that tactile stimulation is a potent driver of performance, potentially reducing response times by up to 34% compared to visual cues alone [17]. By reintroducing the sense of touch, it may be possible to offset the cognitive and motor load imposed by network delays. However, there remains a significant gap in the literature regarding the efficacy of haptic feedback as a specific countermeasure to network latency in endovascular contexts. This study seeks to address this gap by comparing reaction times (RTs) across various sensory stimuli. By determining whether haptic feedback can significantly reduce RT, we aim to provide a technical foundation for safer, more responsive remote thrombectomy systems. Consequently, this multimodal approuch provides a promising mechanism for augmenting the sensory capabilities of practitioners, particularly older professionals, in high-stakes clinical tasks [18].

## 2. Materials and Methods

A prospective, experimental, cross-sectional study was conducted involving healthy volunteers aged 18 to 65 years. Participants were invited to take part in the study between 1 October 2025 and 30 November 2025 using convenience-sampling methods. Recruitment involved direct engagement, with researchers visiting various workplaces and community areas to invite individuals within the eligible age range to participate in the study. The study included all volunteers except those meeting the exclusion criteria, which were having (1) cognitive impairments or (2) physical conditions affecting reaction to different stimuli and motor responses. Verbal informed consent was obtained from all participants included in the study. After enrolling in the study, participants underwent RT testing in response to various stimuli. These included visual and auditory stimulation, superficial and deep-sensory stimulation, and a combination of visual and superficial sensation stimulation designed to simulate the HF used in robotic surgery. For the purposes of this study, haptic feedback (HF) is operationally defined as the delivery of superficial tactile cues. While haptic feedback traditionally encompasses both kinesthetic and force components, we utilized a simplified tactile model to isolate specific sensory variables and minimize confounding factors associated with complex force–feedback systems. The testing was conducted using an experimental system device, which is described in detail below. The tasks carried out to assess RTs to each type of stimulus were as follows:

- Visual stimulation: Participants actuated a $0.035^{\prime \prime }$ guidewire upon activation of five LED lights.
- Audible stimulation: Participants actuated a $0.035^{\prime \prime }$ guidewire in response to a consistent high-frequency beep sound (4096 Hz) at a sound pressure level of 85 to 90 dBA.
- Superficial sensation stimulation: Participants actuated a $0.035^{\prime \prime }$ guidewire when they felt it move. To eliminate visual input, they were not allowed to look at their hand, and headphones playing uniform music were used to block device sounds.
- Deep sensation stimulation: Participants actuated a $0.035^{\prime \prime }$ guidewire in response to a vibration felt on their non-dominant hand, which rested on the device box. Headphones were worn to eliminate auditory distractions.
- Visual and superficial sensation stimulation: Participants actuated a $0.035^{\prime \prime }$ guidewire when they either observed the LED lights activating or felt the guidewire move. Both stimuli were presented simultaneously, with participants wearing headphones throughout the task.

Participants were instructed to actuate a $0.035^{\prime \prime }$ guidewire as quickly as possible upon perceiving each stimulus. Each stimulus remained active until the guidewire was actuated. Each stimulation test was repeated 11 times for each participant. The first repetition served as an adaptation to the task, while the results from the subsequent 10 repetitions were recorded for analysis. In the experiments, we utilized a portable Sentante haptics system developed to study RT and replicate the necessary and equivalent stimulation in a portable form factor. The system comprised the following components:

1. Microcontroller Unit: The system leveraged STM32F334R8T6, a high-performance Arm® Cortex®-M4 32-bit RISC core operating at 72 MHz, featuring a floating-point unit (FPU), high-speed embedded memory, and a wide range of enhanced I/Os and peripherals (STMicroelectronics, Plan-les-Ouates, Geneve, Switzerland).
2. Light Stimulus Source: The system leveraged five super-bright green LEDs made with gallium phosphide, featuring a power dissipation of 105 mW, a DC forward current of 25 mA, a peak wavelength of 565 nm, and a spectral line half-width of 30 nm (OSRAM, Munich, Germany);
3. Sound Stimulus Source: The system employed a magnetic buzzer transducer with a rated frequency of 4096 Hz, sound pressure level of 85–90 dBA at 10 cm, and rated voltage of 2048 Hz in a ½ duty square wave (Same Sky, 6405 SW Rosewood St., Ste C Lake Oswego, OR, USA);
4. Vibration Stimulus Source: The system included a motor with a nominal power supply speed of 14,500 RPM (minimum 12,000 RPM) and a vibration amplitude of 0.75 g (Vybronics, Bac Giang, Vietnam) and Wenzhou, China).
5. Force Stimulus Source: For this, a $0.035^{\prime \prime }$ guidewire attached to a pop-up spike delivering a force of 2 N (Johnson Electric, Hong Kong, China) was used. The 2 N force was selected to represent the higher end of forces used in modern endovascular robots for safe and effective instrument control [19].

The device employs a real-time custom embeded firmware (HFRT2508.bin), which is designed to handle tasks promptly and efficiently. The application layer running on the embeded firmware manages measurements, control, communication, and result calculations. To ensure precision, all delays in the stimulus generation subsystem are compensated for and measured using the following methods: (a) For light stimuli, electrical signals between stimulus activation and guidewire actuation were compared with instrument readings. (b) For audio and vibration stimuli, an external microphone scanned the response, and its electrical signals were compared to guidewire actuation timings. (c) For force stimuli, an external strain gauge attached to an actuator measured the induced haptic force of the guidewire and actuated it while measuring the event timings. In each case, the measured results were validated against instrument readings. The measurements were conducted using a digital oscilloscope (MSO9104A) with the following specifications: a measurement scale of 0.1 ms and a total error of less than 5 ms. Statistical analysis was conducted using SPSS version 25.0 (SPSS Inc., Chicago, IL, USA). Reaction times (RTs) that deviated by more than 2 standard deviations (SDs) from the group mean were identified as outliers, and the corresponding participants were excluded from the final analysis. This criterion was applied to ensure that the dataset represented a normal RT range through effective removal of data points likely to be influenced by loss of concentration, technical glitches, or external distractions rather than physiological capacity. The Shapiro–Wilk test was utilized to evaluate the normality of data distribution. Continuous variables following a normal distribution were expressed as mean ± SDs. Paired-samples *t*-tests were used to compare RTs across different stimuli, while independent-samples *t*-tests were performed to assess differences in RTs between participant groups. The differences were reported as mean values with 95% confidence intervals (95% CIs). The Pearson correlation test (two-tailed) was applied to analyze correlations among continuous variables. To examine RTs across different age ranges, participants were divided into three age groups: Group 1—18–25 years; Group 2—35–45 years; and Group 3—55–65 years. RTs among these age groups were compared using analysis of variance (ANOVA), followed by Tukey’s post hoc test to identify specific group differences. A *p*-value of <0.05 was considered statistically significant. This study was approved by the Vilnius Regional Bioethics Committee on 2025 September 5 Nr. 2025/9-1709-1150.

## 3. Results

### 3.1. Study Participants

A total of 105 participants were initially enrolled in the study. After outliers were removed, the final analysis included 89 participants, including 50 females (56%) and 39 males (44%), with a mean age of 41 ± 16 years.

### 3.2. Reaction Times for Different Stimuli

**Figure 1:**
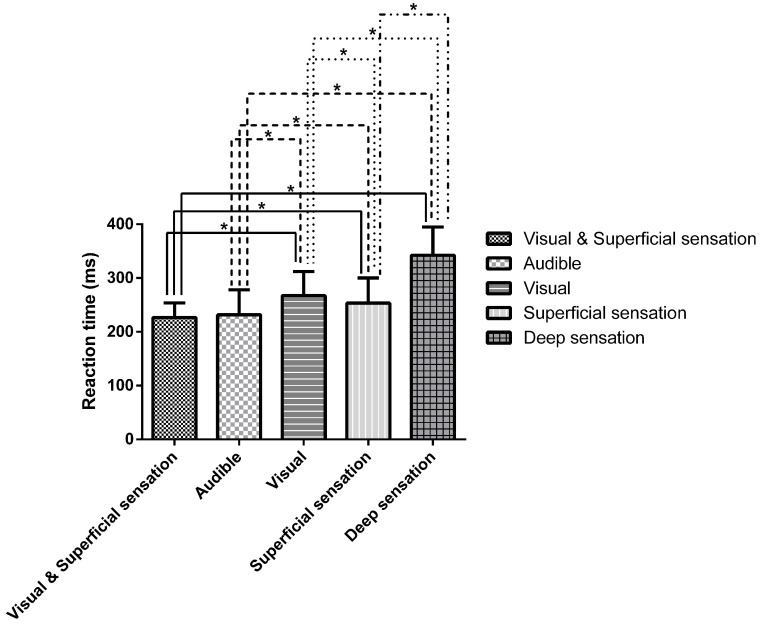
Reaction times for different stimuli (* *p* = 0.001).

The mean RT for combined visual and superficial sensation stimulation was 227 ± 27 ms, which is significantly faster, namely, 40 (95% CI: 32; 48) ms and 26 (95% CI: 20; 33) ms faster, than that for visual and superficial sensation stimulation alone, respectively (both *p* = 0.001) [20]. Additionally, the RT for visual and superficial sensation stimulation was 115 (95% CI: 106; 125) ms shorter than that for deep stimulation (*p* = 0.001), but it was not different from that for audible stimulation (Figure 1).

### 3.3. Age Group Analysis

Participant age showed a significant correlation with RT across various types of stimuli: visual and superficial sensation stimulation (R = 0.54, *p* = 0.001), visual stimulation alone (R = 0.69, *p* = 0.001), superficial sensation stimulation alone (R = 0.56, *p* = 0.001), deep-sensation stimulation (R = 0.55, *p* = 0.001), and audible stimulation (R = 0.49, *p* = 0.001). No significant differences in RTs were found between males and females for any of the tested stimuli (all *p* > 0.05). To further analyze RTs across age subgroups, the participants were categorized into three groups, as described in the methods. Significant differences in RTs across all the tested stimuli were observed among these groups. Post hoc analysis revealed that the participants in the youngest subgroup (Group 1) exhibited faster RTs compared to both Group 2 and Group 3 for all the tested stimuli (Table 1). Middle-aged participants in Group 2 demonstrated faster RTs than those in Group 3 for visual and superficial sensation stimulation as well as deep-sensation stimulation. However, no significant differences were found between Group 2 and Group 3 for RTs for visual stimulation, superficial sensation stimulation, or auditory stimulation (Table 2).

**Figure 2:**
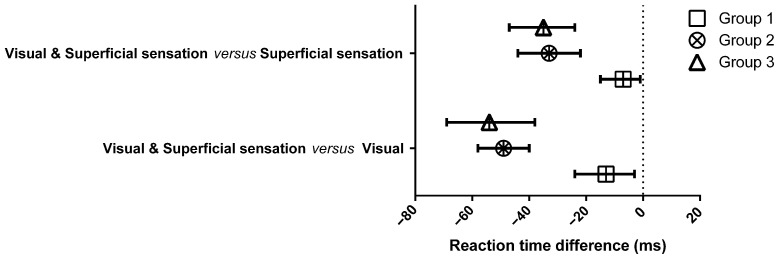
Reaction-time differences between combined visual and superficial sensation and visual and superficial sensation alone.

Additionally, subgroup analysis demonstrated that the RTs for combined visual and superficial sensation stimulation were significantly faster than the RTs for visual stimulation alone or superficial sensation stimulation alone across all three age groups Figure 2.

## 4. Discussion

This cross-sectional experimental study aimed to evaluate whether HF could reduce reaction time (RT) compared to visual and other types of stimulation alone. Our findings clearly demonstrate that combined visual and superficial sensation stimulation, simulating HF used in robotic surgery, significantly reduced RTs by 40 ms (95% CI: 32–48 ms) compared to visual stimulation alone. Furthermore, the effectiveness of HF was consistent across all age groups, including younger participants (18–25 years), middle-aged participants (35–45 years), and older participants (55–65 years), and even increased in older participants. Moreover, the findings revealed that HF enhanced RTs compared to both superficial and deep-sensation stimulation but did not provide an improvement over auditory stimulation. These findings are consistent with prior studies indicating that haptic and auditory stimuli evoke faster RTs than visual stimuli alone and that multisensory integration further enhances the speed of physiological responses [21,22]. The observed benefits of combined stimulation are likely due to multisensory integration in the brain. Visual, auditory, and haptic inputs are processed together in regions such as the superior colliculus and parietal cortex, enabling quicker and more effective sensorimotor responses [23]. Multisensory input improves the efficiency of neural processing, reduces the duration of motor planning, and increases the likelihood of rapid, accurate responses—mechanisms that are particularly crucial for surgical performance under latency conditions. A recent meta-analysis summarized the current evidence and clearly showed that HF in robotic surgery can effectively decrease both average and peak forces applied during surgery, reduce operation time, and improve accuracy and success rates during surgical tasks [24]. Despite a growing body of evidence about the benefits of HF, only a few robotic systems, equipped with HF technology, including Da Vinci 5 (Intuitive Surgical, Sunnyvale, CA, USA) and Sentante (Inovatyvi Medicina, Kaunas, Lithuania), are applied for telesurgery [15,16]. As mentioned previously, latency is one of the most important technical obstacles that should be overcome before wide telesurgery adoption. To date, there are no standardized latency requirements, but it is known that surgeon performance declines exponentially as latency increases, with a threshold of 100 to 200 ms generally considered optimal to avoid compromising surgeon performance [8,9,10]. Naturally, this threshold varies depending on the type of procedure. For instance, in complex laparoscopic surgeries, performance starts to decline at latencies as low as 100 ms [10], whereas up to 400 ms may still be acceptable for endovascular procedures [11,12,13]. The present study demonstrated that HF reduced RT by 40 ms (95% CI: 32–48 ms) compared to visual stimulation alone. Considering that a 70 ms delay is generally unavoidable due to the encoding and decoding of information, and 100 ms is already the threshold for complex surgical procedures [10], the HF-driven RT reduction could be a critical factor in ensuring the suitability of robotic platforms for demanding surgical applications in the context of telesurgery [14]. Furthermore, our experiments demonstrated that the benefit of HF in reducing RT becomes more pronounced in older populations. Given that RTs for visual stimuli generally slow with age [25], this enhanced advantage of HF for older individuals could play a vital role in enabling senior surgeons, who are often highly experienced and skilled, to effectively utilize robotic platforms for telesurgery. Another interesting finding of this study pertains to the similar RTs observed for HF mimicking visual and superficial sensory stimuli as well as auditory stimuli. This aligns with fundamental physiological principles related to motor-evoked potentials. However, the integration of auditory stimulation into current robotic surgery systems requires further investigation [17,26,27,28,29,30,31]. In summary, this study demonstrates the advantage of HF in reducing RT compared to conventional visual stimulation. A particularly compelling finding of this study is that the performance-enhancing effects of haptic feedback (HF) were not only preserved but significantly amplified in older populations. While it is well-established that reaction times (RTs) for visual stimuli typically undergo a linear decline as a function of age—a trend corroborated by the slower baseline RTs in our 55–65 age cohort—the integration of superficial tactile cues effectively narrowed this latency gap. From a neuro-ergonomic perspective, this suggests that multisensory integration provides a more robust pathway for motor-evoked potentials in senior professionals compared to unimodal visual processing. In the high-stakes context of telesurgery, this carries profound implications for professional longevity. Senior surgeons often possess unparalleled clinical judgment and technical mastery, yet their ability to navigate the digital “bottleneck” of network latency may be disproportionately affected by age-related increases in response thresholds. Our results demonstrate that HF can reduce RTs by 40 ms (95% CI: 32–48 ms), potentially offsetting the cognitive and motor load imposed by the unavoidable 70 ms encoding delays inherent in remote systems. While human RT and technical communication latency are fundamentally different phenomena, the observed 40 ms reduction in RT reveals potential for human optimization. By improving the speed of the operator’s response, HF may help mitigate the cumulative delay, although it does not reduce network latency itself. By leveraging tactile–visual co-activation, robotic platforms can effectively “age-proof” the surgical interface, ensuring that the wealth of experience held by older practitioners remains accessible and safe in an increasingly remote operative landscape. However, several limitations of this study must be acknowledged. First, beyond age, we did not evaluate other potential confounders that might influence RT, such as participants’ hobbies, underlying comorbidities, or hand dominance. As a result, it is possible that HF may be less effective in certain subgroups of the population that were not studied. Future studies should incorporate a more detailed participant profile to control for these variables. Second, the experiments utilized very low-amplitude vibrations, which led to notably slower RTs for deep sensory stimulation compared to other stimuli. We chose a vibration amplitude that was similar to that found in the haptics of a mobile device and avoided investigating too deep of a sensation in order to avoid diminishing perception. Higher-amplitude vibrations might produce different results. Third, while this study focused on visual stimulation combined with superficial sensory stimuli equal to a force of 2 N to mimic HF, which aligns with findings showing that this amplitude is critical for providing practitioners with accurate haptic cues during remote cardiovascular interventions [19], other combinations of stimuli were not tested. It is possible that alternative stimulus combinations could be even more effective in reducing RT. Finally, the experiments were conducted on healthy volunteers rather than a population of surgeons. Surgeons may have different RTs in response to various stimuli, meaning that the current study population may not accurately represent the surgeon community. Future research on HF’s impact on RT for the development of surgical robots should prioritize studying surgeons as the primary population. Additionally, experimental studies involving surgeons performing diverse surgical tasks under varying latency conditions, both with and without HF, are essential to better understand HF’s practical applications in clinical settings. In addition, future research could explore integrating novel wearable or other interface systems to establish whether advanced haptic interfaces could improve operators’ performance [32]. Finally, simple trigger-based responses were used in the experiment. These do not accurately replicate the control interfaces used in real surgical robots, limiting ecological validity, so future studies should focus on employing more realistic surgical control simulations.

## 5. Conclusions

This cross-sectional experimental study demonstrates that combining visual and superficial sensation stimulation, mimicking the HF used in robotic surgery, significantly reduces RT by 40 ms (95% CI: 32–48 ms) compared to visual stimulation alone. Additionally, the effectiveness of this combined stimulation appears to be greater in older populations.

## Figures and Tables

**Table 1:** Reaction times for different stimuli across the study groups.

Reaction Times for Different Stimuli	Group 1 (n = 26)	Group 2 (n = 31)	Group 3 (n = 32)	*p* Value
Mean reaction time for visual and superficial sensation stimulation, ms (SD)	207 (15)	224 (14)	244 (32)	*p* = 0.001
Mean reaction time for visual stimulation, ms (SD)	221 (25)	274 (21)	298 (43)	*p* = 0.001
Mean reaction time for superficial sensation stimulation, ms (SD)	215 (17)	258 (32)	279 (54)	*p* = 0.001
Mean reaction time for deep-sensation stimulation, ms (SD)	308 (34)	332 (35)	379 (56)	*p* = 0.001
Mean reaction time for audible stimulation, ms (SD)	192 (20)	244 (37)	251 (50)	*p* = 0.001

**Table 2:** Reaction-time differences between the study groups.

		Visual + Superficial Sensation	Visual Stimulus	Superficial Sensation	Deep Sensation	Audible Stimulus
Group 1 vs. Group 2	Difference, ms (95%; CI)	−17 (−31; −2)	−53 (−73; −32)	−42 (−67; −18)	−23 (−51; 4)	−52 (−77; −27)
	*p*-value	0.017	0.001	0.001	0.109	0.001
Group 1 vs. Group 3	Difference, ms (95%; CI)	−36 (−50; −21)	−76 (−97; −56)	−64 (−88; −39)	−70 (−97; −42)	−58 (−83; −34)
	*p*-value	0.001	0.001	0.001	0.001	0.001
Group 2 vs. Group 3	Difference, ms (95%; CI)	−19 (−32; −5)	−23 (−43; −4)	−21 (−45; 1.7)	−46 (−72; −19)	−6 (−29; 17)
	*p*-value	0.004	0.013	0.075	0.040	0.810

## Data Availability

The raw data supporting the conclusions of this article will be made available by the authors on request.

## Abbreviations

The following abbreviations are used in this manuscript:

RT	Reaction time
HF	Haptic feedback
